# Elemental changes in tooth hard tissues during demineralization and fluoride-induced remineralization: an SEM-EDX study

**DOI:** 10.3389/abp.2026.16706

**Published:** 2026-06-09

**Authors:** Svanishvili Nino, Mamaladze Marina, Mauro Labanca, Vadachkoria Dea

**Affiliations:** 1 Department of Odontology, Tbilisi State Medical University, Tbilisi, Georgia; 2 Dental Clinic, Training and Research Center UniDent, Tbilisi, Georgia; 3 University of Brescia, Brescia, Italy

**Keywords:** demineralization, elemental composition, fluoride, remineralization, SEM-EDX

## Abstract

**Background:**

Tooth enamel and dentin are the most highly mineralized tissues in the human body, and their structural stability depends on the balanced content of calcium (Ca), phosphorus (P), and fluoride (F). Demineralization leads to the loss of these ions, weakening the tooth structure and increasing susceptibility to caries.

**Objective:**

This study aimed to evaluate elemental changes in Ca, P, and F in intact, demineralized, and remineralized teeth across the enamel, dentin–enamel junction and peripulpal dentin.

**Materials and methods:**

45 extracted human teeth were longitudinally sectioned to yield 90 paired specimens. One half of each tooth served as a baseline (intact) control, while the corresponding half was subjected to acid-induced demineralization. Following demineralization, the experimental halves were randomly assigned to one of three remineralization protocols (n = 15 per group): fluoride varnish, fluoride toothpaste with rinsing, and fluoride toothpaste without rinsing. Elemental composition was analyzed using scanning electron microscopy with energy-dispersive X-ray spectroscopy (SEM-EDX). Statistical analysis included paired t-tests for within-tooth comparisons and one-way ANOVA for treatment effects (p < 0.05).

**Results:**

At baseline, enamel exhibited significantly higher calcium and phosphorus levels compared to dentin regions. Demineralization resulted in a significant reduction in mineral content, particularly in dentin, along with complete loss of detectable fluoride (p < 0.0001). Remineralization increased calcium and fluoride levels across all groups. Fluoride varnish showed the highest fluoride retention trend, particularly in enamel; however, no statistically significant differences were observed among treatment groups for calcium and phosphorus.

**Conclusion:**

Fluoride-based treatments contributed to partial remineralization of tooth hard tissues. Fluoride varnish showed a tendency toward greater fluoride incorporation, particularly in enamel; however, statistically significant superiority over other treatment approaches was not demonstrated. These findings support the role of fluoride in remineralization while highlighting the need for further clinically relevant studies.

## Introduction

Tooth hard tissues—enamel and dentin—are the most highly mineralized structures of the human body. Their mineral composition forms the basis of structural strength and functional integrity. Enamel contains approximately 95%–97% inorganic components, primarily hydroxyapatite crystals (Xu et al., 2022; Ten Cate, 2013). In contrast, dentin consists of about 70% mineral content and plays a crucial role in the biomechanical protection of the tooth. Specifically, dentin distributes mechanical loads, shielding the enamel—which, despite its strength, is brittle and prone to fracture—from damage, while also protecting the dental pulp from mechanical and thermal insults (Pashley et al., 2004). Disruption of mineral balance in dental hard tissues is influenced by multiple factors, including nutritional status, personal hygiene, the composition of biofilm, activity of microbiota, and systemic medical conditions such as salivary gland dysfunction or metabolic disorders. Alterations in the balance of key minerals—particularly calcium, phosphorus, and fluoride—initiate demineralization processes, first manifesting as microscopic injuries in enamel and, over time, potentially progressing to dentin. Additional contributors to demineralization include acidic environments, frequent exposure to acidic beverages or foods (e.g., carbonated drinks, citrus fruits), prolonged use of certain medications, hormonal imbalances, and age-related changes that impede normal remineralization processes. Given the structural and chemical complexity of dental hard tissues, effective restoration of mineral balance and caries prevention often require targeted interventions that address both local and systemic influences on mineral homeostasis (Fejerskov and Kidd, 2015; Moynihan and Petersen, 2004; Featherstone, 2008).

The mineral balance in teeth is closely associated with the pH of the oral cavity. A low pH environment promotes the decrystallization of minerals from the enamel and dentine (Margolis and Moreno, 1990). Restoration of this balance is possible only under conditions that provide sufficient ions to support remineralization, such as Ca^2+^, PO_4_^3-^, and F (Xu et al., 2022). Fluoride plays a central role in this process by strengthening the enamel through the formation of fluoroapatite (Ca_5_(PO_4_)_3_F), which is more resistant to acidic challenges and inhibits the metabolic activity of cariogenic microorganisms (Buzalaf et al., 2011; Silva et al., 2020). Consequently, fluoride-containing therapeutic materials, including fluoride varnishes, are among the most effective preventive interventions for caries (Amaechi and Higham, 2005; Svanishvili et al., 2024).

Caries pathogenesis is multifactorial, involving the microbial composition of dental biofilms, dietary habits, saliva properties, and oral hygiene practices (Hicks et al., 2004). Although these factors directly influence the enamel, literature reviews confirm that caries resistance is not determined solely by enamel properties. The structural and chemical characteristics of dentin, particularly the reactivity of specific zones, play a crucial role in the demineralization–remineralization balance of the entire tooth (Pashley et al., 2004; Goldberg et al., 2011; Mount and Hume, 2005; Lussi and Jaeggi, 2008).

Therefore, evaluating caries resistance requires an analysis beyond the inorganic composition of the enamel. The focus of research must include critical dentin regions, such as the dentin–enamel junction and peripulpal dentin. These areas exhibit unique physiological and embryological characteristics, and the collagen matrix of dentin is responsive to ionic changes, underscoring the need for a comprehensive approach to assess tooth mineral dynamics (Pashley et al., 2004; Goldberg et al., 2011).

Modern studies of dental hard tissues increasingly employ scanning electron microscopy and energy-dispersive X-ray spectroscopy (SEM-EDX), which allow precise element analysis and detection of alterations in mineral distribution in different locations (Camargo et al., 2014; Cuy et al., 2002). Our previous research demonstrated significant variations in calcium, phosphorus, and fluoride concentrations across different tooth regions (Svanishvili et al., 2024; Svanishvili et al., 2023). In recent decades, notable clinical advances have been made in caries prevention through the development of therapeutic agents. Prior to clinical implementation, these agents undergo extensive experimental and laboratory testing. Therefore, the creation of artificial caries models employed to study the structure of tooth hard tissues and the effectiveness of treatment and preventive materials is an essential component of cariology research (Petersen and Kavanagh, 2014; Borges, 2011; Zero et al., 2009). Historically, early caries models involved chemically (by the action of chemical agents on the mineral network of tooth hard tissues) or mechanically induced defects in tooth hard tissues. Such approaches produce histological samples without mineral imbalance, or the observed changes are attributed to alternative, involutional origins. Over time, the ideal model for studying caries has become chemically demineralized teeth, primarily induced by organic and inorganic acids. Consequently, teeth with mechanical damage have been excluded from scientific investigations as models of carious lesions.

Developing an effective caries model is challenging because it must account for the cyclical action of cariogenic factors, exposure time, agent specificity, and remineralization intensity. Therefore, the search for a sophisticated and comprehensive experimental model of caries is gaining momentum. Nevertheless, the fundamental principle remains the controlled breakdown and demineralization of hydroxyapatite in the teeth (Zero, 1999; White, 2009; Aldhaian et al., 2019). An analysis of research papers in the field of cariology revealed three primary methods employed for experimental artificial demineralization (Dohan et al., 2017; Moussa et al., 2020; Ccahuana -Vásquez et al., 2019):

1. Chemical–application of acidic solutions to induce dissociation of enamel or dentin minerals;
2. Biological–use of cariogenic bacteria to initiate caries-like processes;
3. Physical–exposure of tooth structures to pH variations, temperature changes, lasers, or ultrasound.

All three methods are suitable for *in vitro* and *ex vivo* experimentation.

It is not surprising that the search for conditions and methods that stimulate or inhibit remineralization has always been a subject of special attention among dentists. Maintaining the integrity of the tooth surface and selecting the best among the hundreds of pharmacological agents available on the market to achieve this goal will be possible only if the doctor (researcher) knows the essence and anatomy of the demineralization process. Since demineralization is based on changes in the mineral components in the structure of enamel and dentin, the study of the dynamics of these microelements will shed light on the problem at hand.

Considering the above, the focus of our research was the analysis of calcium, phosphorus, and fluoride content in three distinct regions of dental hard tissues under three different conditions: intact, demineralized, and remineralized. The originality of our study lies in its investigation of mineral dynamics, not only in the enamel but also at the dentin–enamel junction and in the peripulpal dentin. This comprehensive approach allows for a deeper understanding of the mechanisms underlying caries development and prevention (Svanishvili et al., 2024; Svanishvili et al., 2023; Zero, 1999; Bagramian et al., 2009).

To achieve this goal, we formulated the following objectives:

- Determine the distribution of microelements in the enamel and dentin of extracted human teeth using X-ray spectral analysis.
- The caries process should be modeled *ex vivo* on extracted teeth, and the target microelements in enamel and dentin should be assessed under these conditions.
- Evaluate the effects of three different remineralization methods on the study samples and monitor changes in microelement concentrations.

## Materials and methods

To determine the concentration of target microelements and assess their changes, forty-five extracted, intact human teeth were collected for analysis (The sample size (n = 45) was based on the availability of intact extracted teeth, ensuring adequate representation across the three study conditions (intact, demineralized, and remineralized). Extractions were performed owing to trauma or clinical indications from orthodontists or periodontists. The patients were aged 16–60 years. Only teeth with intact crowns were included in the study. Teeth were stored and handled following the national protocol, “Prevention of infection when handling human extracted teeth, periodontal tissue biopsy, and surgical material” (2020.21.02, #01-282/o). The study protocol on which the submitted article is based was reviewed and approved by the Tbilisi State Medical University Research Ethics Committee on September 23, 2023, at meeting #6-2022/99.

**FIGURE 1:**
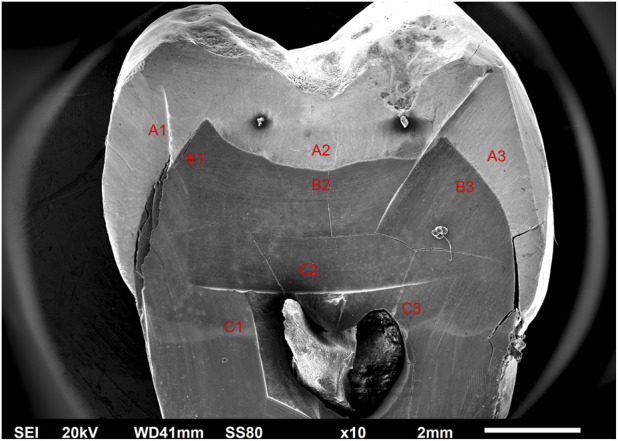
SEM-EDX analysis of tooth hard tissues. Zone A corresponds to enamel, Zone B to the dentin–enamel junction (DEJ), and Zone C to peripulpal dentin. Three regions of interest (A1–A3, B1–B3, C1–C3) were analyzed within each zone to assess elemental distribution (calcium, phosphorus, fluoride) and spatial variability.

As previously described, the concentrations of calcium (Ca), phosphorus (P), and fluoride (F) were assessed in three regions: enamel (zone A), dentin–enamel junction (zone B), and peripulpal dentin (zone C) (Figure 1).

A total of 45 teeth were sectioned longitudinally (bucco-lingually) under continuous water cooling to prevent thermal effects, yielding 90 specimens in total. These were treated as paired samples, where one half of each tooth served as the baseline (intact) control, while the corresponding half served as the experimental counterpart for subsequent demineralization. To ensure accurate within-tooth tracking of elemental changes, specimen identification numbers were strictly preserved throughout all experimental stages. All sections (∼1.5 mm) were standardized, ultrasonically cleaned in deionized water, and polished with a non-fluoride abrasive paste. For the baseline analysis, the 45 intact halves were first treated with 35% phosphoric acid (Ultra-Etch, Ultradent Products Inc., South Jordan, UT, USA) according to the manufacturer’s protocol, then washed and dried.

To prevent surface charging during SEM-EDX analysis, the specimens were coated with a 10 nm gold layer using a vacuum coater (JEC-3000FC, JEOL Ltd., Tokyo, Japan). Elemental composition was determined using a JEOL GSM6510LV scanning electron microscope (JEOL Ltd., Tokyo, Japan) equipped with an energy-dispersive X-ray spectrometer (X-Max, Oxford Instruments, Abingdon, UK) at a working distance of 15 mm. The analysis utilized characteristic X-ray radiation to determine elemental concentrations (mass and atomic percentages). Results were processed and tabulated, including X-ray emission spectra for the targeted areas.

In the experimental phase, the corresponding paired halves (n = 45) were subjected to an *ex vivo* caries model using a cyclic pH-cycling protocol (Delbem et al., 2004; Svanishvili et al., 2025). Solutions were prepared in the laboratory of the LEPL Levan Samkharauli National Forensics Bureau (Tbilisi, Georgia):

- Demineralization solution (pH 4.3): 2.0 mmol/L Ca^2+^ and 2.0 mmol/L PO_4_^3-^ were dissolved in 0.075 mmol/L acetate buffer.
- Remineralization solution with neutral pH (pH 7.0, artificial saliva): 1.5 mmol/L Ca^2+^, 0.9 mmol/L PO_4_^3-^, and 150 mmol/L KCl diluted in 0.1 mol/L Tris buffer.

For 10 days, samples were subjected to a cyclic regimen. For 5 days (Monday to Friday), samples from the study group were placed for 6 h daily (11:00–17:00) in acetate buffer (5 mL per sample), followed by 18 h (17:00–11:00) in a neutral solution (Tris buffer). On weekends, the samples remained in a neutral solution. The solutions were refreshed on the sixth day, and the cycle was continued for another 5 days. Afterward, in the study group samples (in the control group samples as well), microelement concentrations were reassessed in the three regions (enamel (zone A), dentin–enamel junction (zone B), and peripulpal dentin (zone C)) using SEM-EDX.

In the third stage, the study group was subdivided into three subgroups (α, β, and γ; 15 samples each) for remineralization therapy:

- α subgroup: Treated with fluoride varnish Enamelast (Ultradent Products Inc., South Jordan, UT, USA) daily for 10 days, applied for 3 min, and then stored in neutral buffer.
- β subgroup: Teeth were treated twice daily with Opalescence Fluoride Toothpaste (Ultradent Products Inc., South Jordan, UT, USA) for 3 min, lightly rinsed, and stored in neutral buffer.
- γ subgroup: Treated identically to the β subgroup but without rinsing and stored in simulated saliva (Tris buffer).

Following the remineralization stage, all samples were reassessed using SEM-EDX in zones A–C. The resulting data were compared to evaluate the changes in microelement concentrations across the experimental conditions.

### Statistical analysis

Data distribution was assessed using the Shapiro–Wilk test. For comparisons across three conditions (intact, demineralized, and remineralized), one-way ANOVA was applied, followed by Tukey’s *post hoc* test for multiple comparisons. Paired comparisons between corresponding specimen halves (intact vs. demineralized) were performed using paired t-tests. Statistical significance was set at p < 0.05. All analyses were conducted using IBM SPSS Statistics (version 26, IBM Corp., USA).

## Results

### Baseline elemental distribution (control group)

In the control group, elemental analysis revealed a distinct distribution pattern of calcium (Ca), phosphorus (P), and fluoride (F) across the three examined dental zones. Enamel (zone A) demonstrated the highest mineral content, with mean calcium levels of 27.51 ± 3.97, compared to dentin–enamel junction (zone B: 9.01 ± 7.25) and peripulpal dentin (zone C: 9.99 ± 4.56). A similar trend was observed for phosphorus, which was highest in enamel (13.63 ± 1.24) and lower in zone B (6.74 ± 2.81) and zone C (6.70 ± 3.08). Fluoride concentrations were uniformly low across all zones, with values ranging from 0.044 ± 0.032 to 0.055 ± 0.049, indicating minimal natural fluoride content in dental hard tissues. Baseline elemental composition is presented in Table 1.

**TABLE 1:** Baseline elemental composition of calcium (Ca), phosphorus (P), and fluoride (F) in enamel, dentin–enamel junction, and peripulpal dentin (control group).

Zone	Ca (mean ± SD)	P (mean ± SD)	F (mean ± SD)
A (Enamel)	27.51 ± 3.97	13.63 ± 1.24	0.048 ± 0.043
B (DEJ)	9.01 ± 7.25	6.74 ± 2.81	0.044 ± 0.032
C (dentin)	9.99 ± 4.56	6.70 ± 3.08	0.055 ± 0.049

### Effect of demineralization

Following the demineralization procedure, a marked reduction in mineral content was observed, particularly in dentin regions. Calcium levels in enamel showed only a slight decrease (25.20 ± 4.80), whereas a more pronounced reduction was detected in dentin–enamel junction (6.18 ± 3.13) and peripulpal dentin (6.01 ± 2.90).

Phosphorus levels also significantly decreased across all zones, with the most notable reduction in dentin–enamel junction (4.45 ± 2.97) and peripulpal dentin (5.13 ± 2.10), while enamel exhibited a smaller decrease (8.90 ± 4.05).

Fluoride was completely undetectable in all demineralized samples, indicating its loss during the acidic challenge.

Overall, paired statistical analysis demonstrated a statistically significant reduction in Ca, P, and F levels after demineralization across all zones (p < 0.0001). Changes following demineralization are summarized in Table 2.

**TABLE 2:** Changes in elemental composition (Ca, P, and F) in enamel, dentin–enamel junction, and peripulpal dentin following *in vitro* demineralization compared to baseline (paired analysis).

Zone	Ca intact	Ca demin	p	P intact	P demin	p	F intact	F demin	p
A	27.51 ± 3.97	25.20 ± 4.80	<0.0001	13.63 ± 1.24	8.90 ± 4.05	<0.0001	0.048 ± 0.043	0	<0.001
B	9.01 ± 7.25	6.18 ± 3.13	<0.0001	6.74 ± 2.81	4.45 ± 2.97	<0.0001	0.044 ± 0.032	0	<0.001
C	9.99 ± 4.56	6.01 ± 2.90	<0.0001	6.70 ± 3.08	5.13 ± 2.10	<0.0001	0.055 ± 0.049	0	<0.001

### Effect of remineralization treatments

After remineralization, all three treatment approaches (fluoride varnish, fluoride toothpaste with rinsing, and fluoride toothpaste without rinsing) resulted in partial restoration of mineral content.

### Calcium

Calcium levels increased across all zones in all experimental subgroups. However, no statistically significant differences were observed between the three remineralization strategies (p > 0.05).

### Phosphorus

Phosphorus concentrations showed partial recovery in enamel across all treatment groups, while changes in dentin regions remained limited. No significant differences were detected among the three subgroups (p > 0.05).

### Fluoride

Fluoride content increased after all remineralization protocols, with the highest values observed in the fluoride varnish subgroup, particularly in enamel (0.316 ± 0.188). Toothpaste-based treatments showed lower fluoride incorporation, with slightly higher retention observed in the no-rinse subgroup.

Statistical analysis demonstrated a significant difference in fluoride levels in enamel between treatment groups (p < 0.05), while differences in dentin regions were not statistically significant (p > 0.05). Comparisons among remineralization treatments are shown in Table 3.

**TABLE 3:** Comparison of calcium, phosphorus, and fluoride levels across different remineralization treatments (fluoride varnish, fluoride toothpaste with rinsing, and fluoride toothpaste without rinsing) in enamel, dentin–enamel junction, and peripulpal dentin.

Ca	α (Varnish)	β (Toothpaste rinse)	γ (No rinse)	p
A	28.90 ± 4.70	29.48 ± 4.99	29.46 ± 3.25	>0.05
B	6.08 ± 4.44	10.28 ± 7.74	10.10 ± 2.86	>0.05
C	9.18 ± 7.64	11.23 ± 3.60	11.12 ± 4.73	>0.05
P	​	​	​	>0.05
A	13.50 ± 1.73	13.60 ± 1.37	13.99 ± 0.99	>0.05
B	3.11 ± 1.62	4.68 ± 3.13	4.69 ± 1.08	>0.05
C	4.83 ± 2.92	5.22 ± 1.54	5.08 ± 1.59	>0.05
F	​	​	​	​
A	0.316 ± 0.188	0.088 ± 0.050	0.129 ± 0.088	<0.05
B	0.131 ± 0.176	0.099 ± 0.089	0.077 ± 0.061	>0.05
C	0.365 ± 0.391	0.173 ± 0.459	0.203 ± 0.168	>0.05

### Overall interpretation

The results demonstrate that enamel contains significantly higher baseline mineral content compared to dentin, reflecting its highly mineralized structure. Demineralization caused a pronounced reduction in calcium, phosphorus, and complete loss of fluoride, with dentin being more affected than enamel.

Remineralization partially restored mineral content across all groups, with fluoride varnish showing the highest fluoride retention in enamel, although overall differences in calcium and phosphorus recovery among treatment methods were not statistically significant.

These findings highlight the differential susceptibility of enamel and dentin to demineralization and their variable response to remineralization strategies.

## Discussion

The present study evaluated elemental changes in enamel, the dentin–enamel junction (DEJ), and peripulpal dentin during demineralization and fluoride-mediated remineralization using SEM-EDX analysis. The principal findings demonstrated that enamel exhibited substantially higher baseline calcium (Ca) and phosphorus (P) concentrations than dentin regions, and demineralization caused a significantly greater reduction in mineral content in dentin than in enamel. Furthermore, while fluoride became undetectable following demineralization across all zones, subsequent remineralization partially restored mineral content, with fluoride varnish showing the highest fluoride incorporation in enamel. These findings are relevant because they map tissue-specific profiles of mineral loss and recovery across distinct tooth regions within a single, controlled experimental model, offering a clearer understanding of intra-tooth remineralization dynamics. While the higher baseline concentrations of Ca and P in enamel versus dentin align with the classic structural model of tooth hard tissues (Ten Cate, 2013; Goldberg et al., 2011), our regional mapping introduces a more nuanced perspective. Previous studies often treat dentin as a uniform substrate or focus solely on outer enamel surfaces (Fejerskov and Kidd, 2015; Svanishvili et al., 2024). By analyzing the enamel, DEJ, and peripulpal dentin simultaneously within a paired split-sample design, our data capture a distinct mineral gradient. Specifically, the profound mineral depletion observed at the DEJ and peripulpal dentin highlights that acid susceptibility is highly compartmentalized. This deeper regional vulnerability is often overlooked in traditional setups but matches the structural patterns observed by (Featherstone, 2008; Svanishvili et al., 2023). The complete loss of detectable fluoride across all regions post-demineralization provides critical context to ongoing discussions regarding fluoride stability. While earlier investigations noted rapid fluoride depletion on superficial enamel under acidic challenges (Margolis and Moreno, 1990; Zero et al., 2009), our findings expand this premise by demonstrating that fluoride depletion is not just a surface phenomenon but extends into the deeper dentin architecture. This implies that under severe acid challenges, the structural reservoir of fluoride is depleted comprehensively across the tooth cross-section, accentuating the vulnerability of underlying dentin zones. In the context of remineralization, our data show that while fluoride varnish significantly enhances fluoride uptake in enamel, this superiority does not fully translate to superior Ca and P crystal-lattice restoration across all dentin regions when compared to other protocols. This observation challenges the generalized assumption that higher superficial fluoride accumulation automatically dictates uniform, tooth-wide remineralization efficiency (Buzalaf et al., 2011; Aldhaian et al., 2019). Instead, our findings support the view that the organic matrix of dentin acts as a rate-limiting barrier to mineral precipitation, a phenomenon that warrants closer attention when designing clinical protocols for root caries or deep lesions. Similarly, the slightly higher fluoride retention observed in the non-rinsing toothpaste group, though non-significant, reinforces the kinetic argument made by (Silva et al., 2020) that prolonged ion availability is essential for diffusion past the superficial layers. What is fundamentally new in this study—and what has not been adequately captured in the existing literature—is the simultaneous tracking of elemental fluxes across three distinct topographical zones of the same tooth during sequential structural states. Where previous SEM-EDX studies have typically compared isolated substrates or independent groups (Svanishvili et al., 2024; Svanishvili et al., 2025), our paired design isolates tissue architecture as the primary variable. This contributes direct evidence that the DEJ and peripulpal dentin behave as distinct thermodynamic systems during both acid dissolution and mineral therapy, shifting the focus from simple surface interactions to three-dimensional intra-tooth mineral dynamics. Several limitations should be acknowledged. First, the *ex vivo* design cannot fully replicate the complexity of the oral environment, including salivary buffering, microbial biofilms, and fluctuating pH conditions. Second, the sample size was based on specimen availability rather than *a priori* statistical power analysis. Third, the broad age range of the included samples (16–60 years) was not stratified and may have contributed to variability in mineral composition and fluoride response. Furthermore, although SEM-EDX provides detailed elemental characterization, it does not directly assess structural or mechanical recovery of dental tissues. Future studies should include larger sample sizes, age-stratified analyses, pH-cycling protocols more closely simulating clinical conditions, and complementary methods such as microhardness or micro-CT evaluation (White, 2009; Dohan et al., 2017; Ccahuana-Vásquez et al., 2019). Overall, the present findings demonstrate substantial differences in mineral dynamics between enamel and dentin.

## Conclusion

Within the limitations of this *ex vivo* study, enamel demonstrated greater resistance to demineralization compared to dentin, which showed more pronounced mineral loss particularly at the dentin–enamel junction and peripulpal dentin. Complete fluoride depletion following demineralization suggests its potential utility as a sensitive marker of mineral loss. All three fluoride-based remineralization protocols partially restored mineral content. Fluoride varnish demonstrated a tendency toward higher fluoride incorporation in enamel; however, this did not translate into statistically significant differences in calcium or phosphorus recovery across treatment groups. Therefore, no definitive conclusion regarding the superiority of any single treatment method can be drawn from the present data. Further investigation under clinical conditions with larger, age-stratified samples is warranted to confirm these findings.

## Data Availability

The original contributions presented in the study are included in the article/supplementary material, further inquiries can be directed to the corresponding author.
